# P-942. Infective Endocarditis in Pregnancy: a Retrospective Review of Maternal and Neonatal Outcomes

**DOI:** 10.1093/ofid/ofae631.1133

**Published:** 2025-01-29

**Authors:** Andrew J Robinson, Madeline King, Alexandra Hanretty, Geena Kludjian

**Affiliations:** Cooper University Hospital, Philadelphia, Pennsylvania; Cooper University Hospital, Philadelphia, Pennsylvania; Cooper University Hospital and Cooper Medical School of Rowan University, Philadelphia, Pennsylvania; Cooper University Hospital and Cooper Medical School of Rowan University, Philadelphia, Pennsylvania

## Abstract

**Background:**

Infective endocarditis (IE) in pregnancy has an annual incidence of 0.006%. As rates of injection drug use increase across the US, there is the likelihood that the rates of pregnant people with IE increases. There is a paucity of data of IE in pregnancy and implications on maternal and neonatal outcomes. The purpose of this retrospective chart review was to describe the maternal outcomes of patients who are pregnant with IE and neonatal outcomes from a tertiary care center with a level 3 neonatal intensive care unit (NICU).

**Figure:**
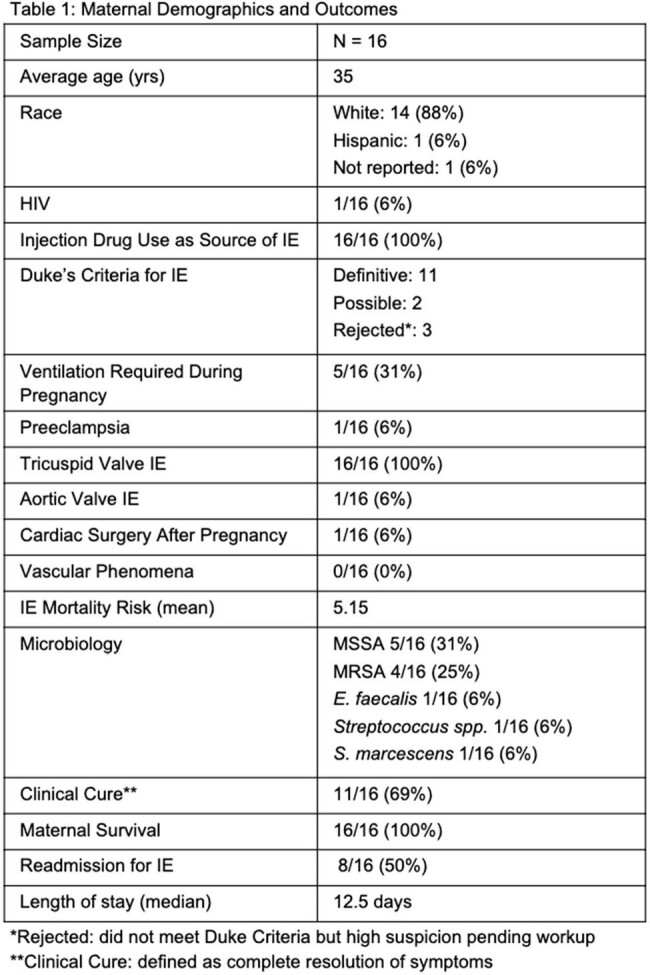

**Methods:**

33 patients were identified as having both IE and pregnancy from January 1, 2017, through January 31, 2022; 16 met inclusion criteria. Data was matched to birth records and 10 neonates were included. Maternal outcomes included survival, clinical cure, complications in pregnancy, type of birth and term of childbirth. Neonatal outcomes included survival, need for NICU admission, treatment for neonatal abstinence syndrome (NAS), and complications at birth including social issues. All maternal and neonatal results are reported in figures 1-3.

**Figure:**
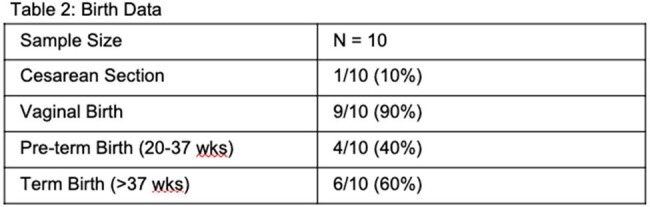

**Results:**

All patients had IE of their tricuspid valve, and 1 had concomitant aortic valve infection. None had valvular surgery during pregnancy, and one had valvular surgery after pregnancy. Preeclampsia occurred in 1 patient. 40% of births were preterm, which is higher than the national average of 10.4%; 10% were cesarean births, 90% were vaginal. 70% of neonates required NICU admission and treatment for NAS, and 40% required respiratory support. The average neonatal length of stay was 39.9 days. 6 babies had delayed discharge due to social issues.

**Figure:**
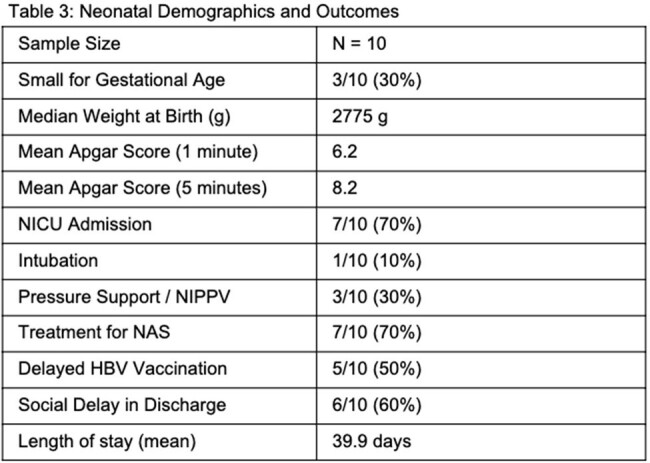

**Conclusion:**

The study demonstrates 100% maternal and neonatal survival, however, conveys significant neonatal morbidity, including need for NICU level of care, increased rates of preterm births, need for treatment for NAS and prolonged hospitalization due to social issues. There are potential health consequences for the neonate, as treatment for NAS has been shown to increase risk for substance use disorder later in life. This study is limited by its small sample size; however, this cohort is one of the larger cohorts to represent this population.

**Disclosures:**

**Madeline King, PharmD**, Innoviva: Advisor/Consultant **Alexandra Hanretty, PharmD**, Abbvie: Advisor/Consultant

